# Flapping-wing robot achieves bird-style self-takeoff by adopting reconfigurable mechanisms

**DOI:** 10.1126/sciadv.adx0465

**Published:** 2025-09-03

**Authors:** Ang Chen, Bifeng Song, Kang Liu, Zhihe Wang, Dong Xue, Hongduo Qi

**Affiliations:** ^1^ School of Aeronautics, Northwestern Polytechnical University, Xi’an, Shaanxi 710072, China.; ^2^ National Key Laboratory of Aircraft Configuration Design, Xi’an, Shaanxi 710072, China.

## Abstract

Flying vertebrates use specialized wingbeat kinematics in hovering, takeoff, and landing, featuring ventrally anterior downstrokes and aerodynamically inactive upstrokes to enhance aerodynamic characteristics at low airspeeds. Rarely implemented in robotics, this inspired RoboFalcon2.0, a flapping-wing robot with reconfigurable mechanisms performing bioinspired flap-sweep-fold (FSF) motion for controlled bird-style takeoff. FSF couples flapping, sweeping, and folding within a single wingbeat cycle, mimicking vertebrate slow-flight kinematics. Wind tunnel tests demonstrate that sweeping amplitude modulates lift and pitching moment in FSF motion. Computational fluid dynamics simulations reveal that FSF’s aerodynamic effects correlate with leading-edge vortex strength and pressure center location. Dynamics simulations analyze pitch control during takeoff. Real-world flights validate RoboFalcon2.0’s self-takeoff capability. This work advances avian-inspired robotics through vertebrate-like actuation principles, enabling more biomimetic flapping-wing designs.

## Teaser

A bird-inspired flapping-wing robot achieves bird-style takeoff locomotion by exploiting reconfigurable mechanisms.

## INTRODUCTION

**Fig. 1.**
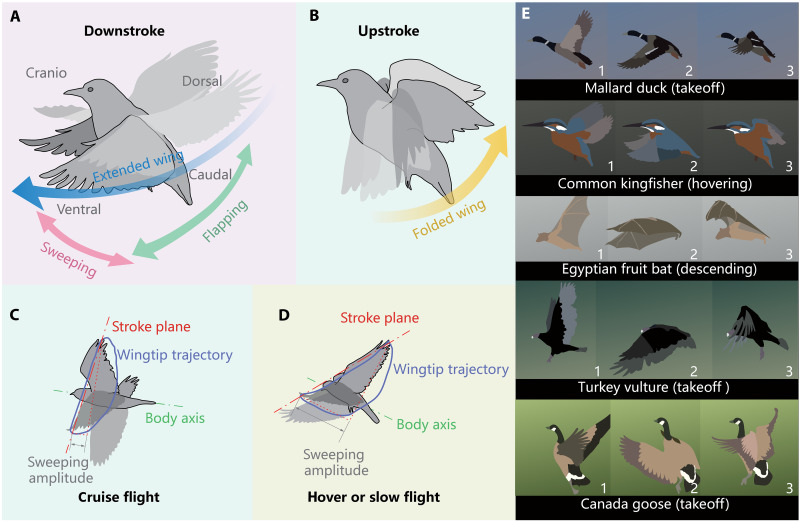
Typical bird wingbeat pattern in slow flight. (**A**) Downstroke of the asymmetrical hovering. The stroke can be considered as a combination of ventral flapping and cranio sweeping, with the wings being kept extended. (**B**) Upstroke of the asymmetrical hovering. The wings are kept flexed during the recovery stroke. (**C**) Stroke plane in cruise flight, steeper relative to the body and horizontal line, and the wing sweeping amplitude is relatively negligible. (**D**) Stroke plane in slower flight, more inclined relative to the body and horizontal line, and the sweeping amplitude is enhanced. (**E**) Slow-flight wingbeat pattern of different species. e.g., takeoff, hovering, and descending.

Flying animals always experience low airspeed situation during flight like hovering, takeoff, and landing. Compared to insects’ hovering flight, which has been commonly observed and discussed, most flying vertebrates tend to adopt more sophisticated wing kinematics for sufficient lift to maintain the flight under low-airspeed condition. As in their cruise flight, birds and bats apply greatly deformed or tucked wings in upstrokes during slow flight, which arises from the fact that their articulated forelimb anatomy allows for having large wing morphing ability (*1*, *2*). Some slow-flying birds and small bats with short rounded wings are considered to apply the aerodynamically inactive upstroke during low-speed flight, which means all aerodynamic lift should be generated by the downstroke (*3*). This aerodynamic inactive upstroke is also adopted by some passerine bird in their untraditional hovering flight, known as asymmetrical hovering (*3*–*6*). This rarely discussed hovering kinematics differs from the symmetrical hovering applied by hummingbirds and insects, where the latter generates lift in both downstroke and upstroke (*6*–*8*). Specifically, in an asymmetrical hovering, the downstroke starts with the wings extended dorsally, and both wings are kept fully extended and sweep both anteriorly and ventrally during the downstroke (Fig. 1A). Then, the wings are flexed during upstroke and return to the dorsal side (Fig. 1B) (*4*, *6*). This hovering mechanism is also observed in some relatively larger birds and bats (*9*–*14*). Overall, the wing kinematics described above creates a forward-leaning stroke plane that is distinguished from the basically horizontal one observed in hummingbirds and insects.

Research suggests that the wingbeat kinematics in flying vertebrates generally changes gradually with increasing flight speed (*15*, *16*). The wingtip stroke plane of birds and bats is more perpendicular relative to the body and horizontal line in faster cruise flight and more inclined in slower flight (*15*–*19*). It is believed that inclined wingstroke would primarily direct airflow downward to support the body weight (*17*, *20*–*22*). Moreover, in hovering and steeper ascending and descending flight, more anterior wing position in downstroke is generally exhibited (*22*). The orientation of the stroke plane in hovering and speed-varying flight may determine the direction of total aerodynamic force, while the angle of stroke plane with respect to the body is affected by the wings’ anterior protracting (*15*, *17*, *22*).

As with the typical slow-flight kinematics of birds illustrated in Fig. 1, for example, the stroke plane is steeper relative to the body in cruise flight, and the wings’ anterior protracting is almost negligible (Fig. 1C). As speed decreases, the stroke plane is more inclined relative to the body, the craniocaudal sweeping increases in amplitude, and the wings are more protracted overall (Fig. 1D). The anteroposterior protracting and retracting of forelimbs are essential for flying vertebrates to adjust the forward and backward sweep angle of their wings. Birds and bats can thereby regulate the relative position of the aerodynamic center and center of gravity (CG), thus having an effect on pitch agility and maneuverability (*2*, *23*). The downstroke of the wingbeat pattern shown in Fig. 1A can be interpreted as a combination of ventral flapping and anterior sweeping, and the upstroke as a combination of dorsal flapping and posterior retracting with folded wings. Depending on whether the upstroke is aerodynamically active or not, the wing morphing and folding patterns during upstroke are left up for debate among species (*16*, *24*). However, downstroke in which the wing remains extended and flapping downward and sweeping forward is widely shared in flying vertebrates. Figure 1E presents five flying vertebrates of different scales to demonstrate the universality of this wingbeat pattern across species. Each species undergoes distinct low-speed flight phases (e.g., takeoff: mallard, turkey vulture, and Canada goose; descending: Egyptian fruit bat; and hovering: kingfisher). Notably, all species exhibit a distinct forward sweep during the downstroke of their wings. The wingbeat slow motion of these illustrated species is also shown in movie S1. In summary, such wingbeat pattern ensures a variation continuity of locomotion form in flying vertebrates during different flight states such as hovering (also including takeoff/landing), slow flight, and cruising. This, in turn, yields an interesting inspiration for flying robots that use flapping-wing flight as their primary form of locomotion.

Anteroposterior wing sweeping has already inspired a number of designs for drones and flying robots. For example, bionic feathered wings with sweeping capability have been applied to some bird-inspired propeller-driven drones, which substantially enhance their rolling and pitching maneuverability (*25*–*27*).Foldable wing based on flying vertebrates’ forelimb anatomy has also been used in some designs for flapping-wing flying robots (*28*–*31*). However, these features are currently used mainly in cruising or gliding flight and have not been discussed more in terms of enhancing the slow flight or takeoff/landing capabilities for flapping-wing flight. Some flapping-wing robots with hovering and self-takeoff capabilities have been designed and investigated (*32*–*34*), but they mostly adopt the wing kinematics inspired by insect- or hummingbird-style symmetrical hovering. The few bird-scale flapping robots with self-takeoff capability, nonetheless, still have to rely on jumping, catapult, or other assistive devices for takeoff launching (*35*–*38*). These insect- and bird-inspired cases exclusively use single degree-of-freedom (DOF) wing kinematics: The wings reciprocally rotate solely about the longitudinal axis of the body, relying on passive aeroelastic torsion induced by aerodynamic loads to generate thrust. This exhibits stark kinematic divergence from the pronounced folding and sweeping motions observed in flying vertebrates during low-speed flight. As for the typical bird slow-flying wing kinematics in Fig. 1 discussed above, it has only been investigated experimentally and simulatively in some cases (*39*–*42*). There is no flapping-wing robot yet that uses the ventral-anterior flapping downstrokes with the aerodynamically inactive upstrokes to accomplish a flight process similar to those observed in birds and bats.

**Fig. 2.**
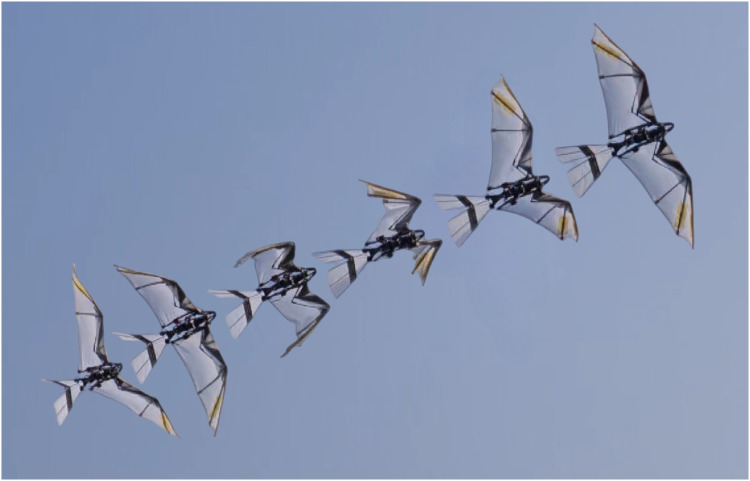
Robotic imitation of bird-inspired cruise flight in our previous work. A reconfigurable actuation strategy has been applied on the flapping-wing robot named RoboFalcon introduced in (*30*).

However, implementing the kinematically variable wingbeat pattern in Fig. 1 on a flapping-wing robot poses a substantial engineering challenge. For flying vertebrates, wing flapping, sweeping, and folding can be regarded simplistically as three DOFs. According to Fig. 1 (A and B), these three motions need to follow the same wingbeat period at different phases to perform a coupled motion, resulting in what we call flap-sweep-fold (FSF) wing motion. This wing motion applied on robots should also feature independently adjustable sweeping and folding to adapt to locomotion forms at various flight speeds or other flight states. Whereas the wingbeat frequency of a bird-scale flapping-wing robot is too high for a lightweight and low-power actuator to drive the inertial components reciprocally, a more responsive and powerful actuator would be too heavy for flight tasks. Assigning a separate actuator for each DOF would cause a contradiction in weight, power, and control bandwidth. Therefore, one feasible idea is to use some sort of reconfigurable mechanism regulated by several low-power actuators to convert the actuation of a single high-power main actuator into the desired wing motion. Our previous research has already presented a reconfigurable mechanism (*30*), known as the conical rocker mechanism (CRM), which is capable of performing the cruise flight wing kinematics inspired by birds and bats. The flapping-wing robot we developed based on this mechanism, RoboFalcon, exhibits wingbeat pattern as shown in Fig. 2. As we discussed earlier, the RoboFalcon can only perform cruise flight but cannot do flight at lower airspeeds with the wingbeat pattern shown in Fig. 1 (A and B).

Here, we present a previously unexplored medium-bird-scale flapping-wing robot design, named RoboFalcon2.0, that is capable of self-takeoff and slow flight using the FSF wing motion. The wing motion is driven by a set of unique reconfigurable actuation mechanisms evolving from our previous research. These mechanisms ensure that the RoboFalcon2.0 can take off and fly forward using the ventral-anterior flapping downstrokes with the tucked upstrokes to generate lift and thrust, namely, the bird-style takeoff. The wing sweeping and folding amplitude can also be tuned for pitch and roll control during flapping. The ability and characteristics of FSF wing motion to generate lift, thrust, and pitching moment are investigated and analyzed using wind tunnel tests and computational fluid dynamics (CFD) methods. The controlled bird-style takeoff process performed using FSF wing motion is discussed through dynamics simulation. A quasi-steady aerodynamic estimation model adapted to FSF wing motion is applied in the simulation. Real-world flight tests are conducted to verify the takeoff and flight control capability of the RoboFalcon2.0. The results show that the bioinspired FSF wing motion can bring certain lift and head-up moment enhancement under both windless and low-airspeed conditions, which helps achieve the pitch trimming in takeoff and slow forward flight. The sweeping amplitude reconfigurability introduced by the actuation mechanisms can function as an effective pitching maneuver strategy for takeoff process of bird-scale flapping robot, which in turn allows RoboFalcon2.0 to perform a controlled bird-style self-takeoff locomotion.

## RESULTS

### Platform and mechanical design

**Fig. 3.**
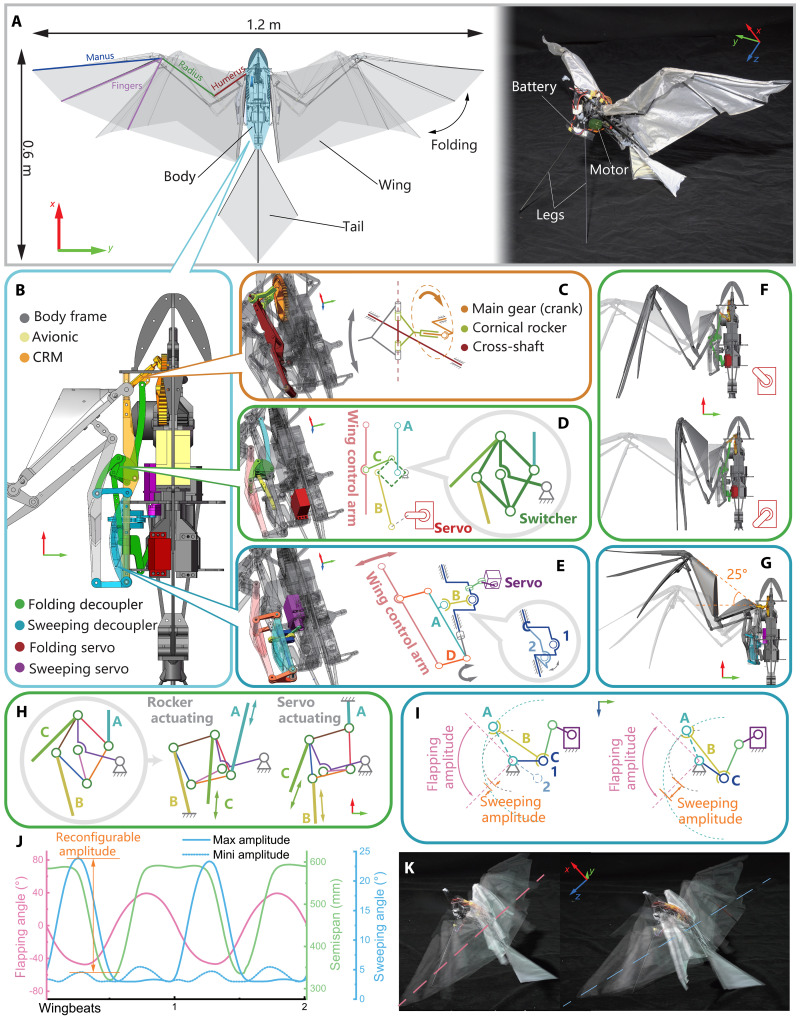
Platform and mechanical design of RoboFalcon2.0. (**A**) RoboFalcon2.0 flapping-wing robot. Top view of the robot with the multilink bat-style wing fully extended and folded (left). Battery and motor layout of the robot, standing on its tail with two carbon fiber rod legs supporting the body (right). (**B**) Mechanical layout of the wing motion actuation system. (**C**) Kinematic diagram of the CRM. (**D**) Kinematic diagram of the folding decoupler. (**E**) Kinematic diagram of the sweeping decoupler. (**F**) Folding decoupler function demonstration. Wing folding is actuated by the conical rocker with the servo driven freed (shown above). Wing folding is controlled by the servo with the conical rocker freed (shown below). (**G**) Sweeping decoupler function demonstration. This mechanism provides the wing with a maximum forward-swept angle about 25°. (**H**) Switcher function demonstration. Folding decoupler function is enabled by switching between rocker actuating and servo actuating. (**I**) Rear view of the sweeping decoupler. Illustration of the state of minimum sweep (left) and maximum sweep (right). (**J**) Variation of kinematic parameters within two wingbeats of the FSF wing motion. Sweeping angles with different amplitudes are distinguished by solid and dashed blue lines. The sweeping amplitude can be reconfigured independently without affecting other parameters. (**K**) Stroke planes of the FSF wing motion with two different sweeping amplitudes, minimum (left) and maximum (right). The enhanced sweeping amplitude creates a more inclined stroke plane, just like the bird wingbeat pattern discussed in Fig. 1.

The RoboFalcon2.0 flapping robot platform we designed (Fig. 3A) is based on the scale of a medium-sized bird such as the peregrine falcon, with a wingspan of 1.2 m and a total weight of nearly 800 g. Its primary characteristics are shown in Table 1.

**Table 1.** Specification of RoboFalcon2.0.

Parameter	Value
Total weight	800 g
Wingspan	1.2 m
Wing loading	3.64 kg/m^2^
Flapping amplitude	85°
Maximum flapping frequency	7.5 Hz

Referring to flying vertebrates, the wing applies a three-segmented skeleton, consisting of the humerus, radius, and manus, covered with polyester fabric membrane to form a bat-style wing. The mechanical kinematics of the skeleton allows the wing to fold spanwisely to change the planform shape and area (Fig. 3A).

The body frame (Fig. 3B, denoted in gray) is made from carbon fiber composite boards, which provide the necessary support for the whole mechanical operation. The machine is driven by a brushless direct current (BLDC) motor, with the power transmitted through a two-stage gear reduction to the CRM (highlighted in orange in Fig. 3B) located at the wing root, which then drives the wing flapping up and down by a cross-shaft hinge (Fig. 3C, denoted in red).

As the CRM and wing mechanical design have been well discussed in our previous work (*30*), we are not going to delve deeply into these two parts in this paper. The major innovation of this work is the design of wing motion decoupling mechanism (Fig. 3B, denoted in green and blue) in collaboration with the CRM, which can transform the rotational motion of the conical rocker (Fig. 3C, denoted in yellow) into a controllable and effective FSF wing motion as mentioned in Introduction. The decoupling mechanism consists of two sets of multilink systems located at wing root, namely, the folding decoupler (Fig. 3D) and sweeping decoupler (Fig. 3E).

#### Folding decoupler

The folding decoupler provides the ability to transform the rotational motion of the conical rocker (Fig. 3C, yellow) into a reciprocating motion along the longitudinal direction via the linkage A (Fig. 3D, light blue), thus enabling the cyclic actuation of the wing’s folding. It also allows the rear-mounted servo with capability to control/limit the maximum wing-expanding range via linkage B (Fig. 3D, yellow).

The squared part where links A and B are articulated together is called the folding switcher (Fig. 3D, dark green), which is a five-link mechanism. The folding actuation is output to the wing (wing control arm; Fig. 3D, in light red) through the folding output linkage C (Fig. 3D, light green) which is hinged onto the folding switcher. The unique design of the switcher’s linkage layout allows wing folding actuation to be swapped between the conical rocker and the servo (Fig. 3D, denoted in red). Specifically, when the conical rocker is running in posterior travel, the wing is actuated to fold by the conical rocker with no control from the servo (Fig. 3F, above). In this case, the state of the switcher linkage system is shown in Fig. 3H (middle, rocker actuating). Linkage C is only acted by linkage A and is almost unaffected by linkage B, which is connected to the servo. When the conical rocker is running in anterior travel, the rocker is no longer actuating the wing to fold, and the control authority is transferred to the servo (Fig. 3F, bottom). In this case, the state of the switcher linkage system is shown in Fig. 3H (right, servo actuating). Linkage C is acted by linkage B and is almost unaffected by linkage A, thus enabling the servo actuating.

On the basis of this arrangement, when the main gear is rotated in the direction shown in Fig. 3C, the wing-folding phase difference always lagged behind wing flapping by 90° (Fig. 3J), which means that the wing is actuated by the conical rocker to fold in during upstroke, and the servo determines whether to maintain a full or limited wingspan during downstroke. This feature is consistent with the mechanical function of the RoboFalcon in our previous study but is implemented differently. The key point is that the redesigned folding decoupler in this paper is compatible with the sweeping motion of the wing.

#### Sweeping decoupler

The sweeping decoupler is able to convert the reciprocating rotation of the flapping shaft due to wing flapping into a reciprocating swing of the sweep-actuating arm A (Fig. 3E, light blue) along the lateral direction by a ball joint link B (yellow in Fig. 3E) connected with a crankshaft C (dark blue in Fig. 3E, coaxial with the flapping shaft) located at the rear of the wing root. The sweep-actuating arm A is connected to the wing control arm (Fig. 3E, light red) via two sweeping output link D (Fig. 3E, orange) to form a four-link mechanism, which can convert the reciprocating swing of the sweep-actuating arm into the anteroposterior reciprocating sweeping motion of the wing as a whole independently from the folding. We use a servo to control the angle of the crankshaft (Fig. 3E, purple). When the crankshaft is in position 1, the amplitude of the sweeping motion during wing flapping is limited to a very small range (less than 5°), which is exhibited by the rear view shown in Fig. 3I (left). When the crankshaft gradually transitions from position 1 to position 2, the amplitude of the sweeping motion is being amplified (Fig. 3G), which is also shown in Fig. 3I (right). For the latter situation, when the main gear drives wing flapping, the wing sweeping motion is synchronized with the flapping motion by a phase lag of 180° (Fig. 3J). Specifically, it sweeps backward to its limit when the wing is lifted to its highest position and forward to its limit when the wing flaps down to its lowest position. This feature synchronizes the periodic sweeping motion with the flapping motion and gives the ability to reconfigure the amplitude of sweeping (5° to 25°). The above mechanical principle and design are quite complicated; refer to the Supplementary Materials (movie S2) for more details.

Overall, the CRM combined with the presented folding and sweeping decoupler can realize the FSF wingbeat pattern as mentioned in Introduction, and it can control the folding and sweeping amplitude of the wing by servos independently (see movie S3). Figure 3K illustrates the tilted stroke planes (indicated with dashed lines) for wingbeat patterns with two different sweeping amplitudes. When the above presented wing modules are arranged symmetrically on the left and right, the RoboFalcon2.0’s entire wing actuation system is equipped with five control DOFs including flapping (see Materials and Methods).

We have already discussed the effect of unilateral wingspan limitation for the folding-coupled flapping on rolling maneuvers in our previous study (*30*). Here, we focus on investigating the longitudinal aerodynamic characteristics of the reconfigurable sweeping amplitude for FSF wing motion.

### Wind tunnel experiment and CFD analysis

**Fig. 4.**
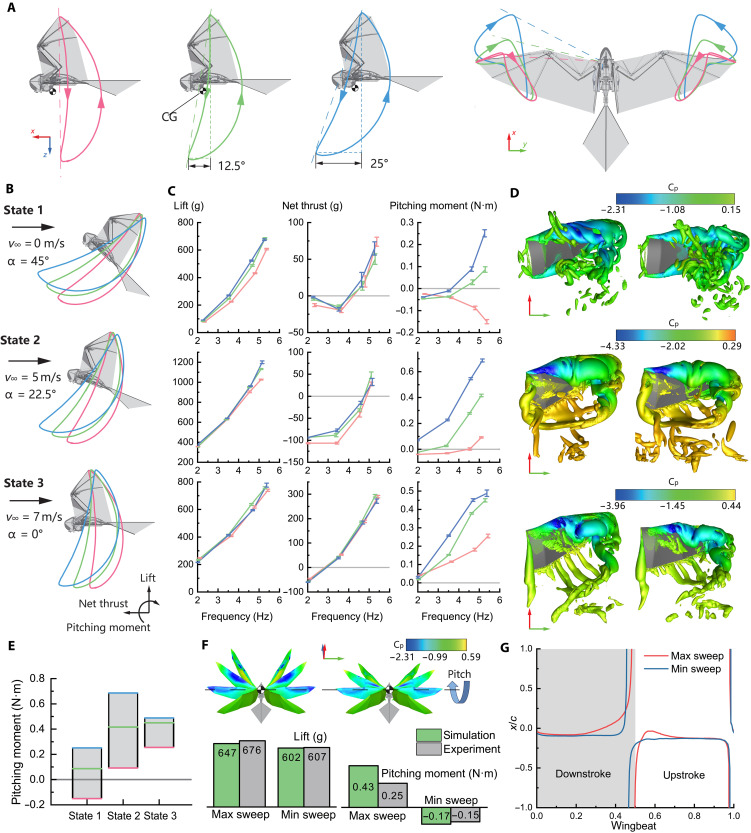
Wind tunnel experiment and CFD analysis for FSF wing motion in different flight states. (**A**) Wingtip trajectories (side view and top view) for the FSF wing motion with three different sweeping amplitudes, minimum (red), medium (green), and maximum (blue). (**B**) Three flight states at different angles of attack (α) and airspeeds (*v*_∞_) for wind tunnel measurement and CFD analysis. The aerodynamic coordinate system is also shown below. (**C**) Measured results of cycle-averaged lift, net thrust, and pitching moment for different flight states (aligned with the flight states on the left and colored with the corresponding wingtip trajectories). Each measurement point is noted with a standard deviation error bar from different flapping cycles. (**D**) Vortex distributions around the wings with maximum (left) and minimum (right) sweeping amplitudes at middle-downstroke moment for three flight states (aligned with the flight states on the left) at the flapping frequency of 5.5 Hz. Vortices are visualized with iso-surfaces based on the *Q* criterion (*Q* = 40,000 for state 1, *Q* = 15,000 for state 2, and *Q* = 25,000 for state 3) and colored with local pressure coefficient (*C*_p_, obtained based on the wingtip velocity). (**E**) Pitching moment varies across the three flight states. (**F**) Surface pressure distributions of the wings with maximum (top left) and minimum (top right) sweeping amplitudes at four downstroke moments for state 1 (from the top view) at flapping frequency of 5.5 Hz. The discrepancies between the experimental and simulated aerodynamic values for this state are illustrated below. (**G**) CP’s normalized location (*x*/*c*) in one flapping cycle for the wings with maximum (red) and minimum (blue) sweeping amplitudes for state 1. The *x/c* stands for the ratio of *x* displacement (CP from CG) to the mean aerodynamic chord length.

We conduct wind tunnel experiment and CFD simulation to study the aerodynamic characteristics of the FSF wing motion with the variation of sweeping amplitude at different flight states. Figure 4A shows the wingtip trajectories of FSF wing motion with three different sweeping amplitudes. The relatively tilted blue trajectory on the right indicates that the sweeping decoupler maximizes the sweeping amplitude (about 25° forward-swept angle), the red trajectory on the left shows that the sweeping amplitude is negligible (flap-fold wing motion only), and the in-between green trajectory represents that the sweeping amplitude is halved (about 12.5°). As discussed in Introduction, the wing kinematics applied on RoboFalcon2.0 are inspired by the flapping pattern of flying vertebrates during low-speed flight (takeoff, landing, and hovering), and the angle of attack will be correspondingly increased to adapt to the lift demand as the flight speed decreases. Based on this and RoboFalcon’s empirical cruising speed (around 8 m/s) in our previous work (*30*), we select the flight states comparable with that from takeoff to cruising as the subjects of this study. Figure 4B illustrates the three flight states being considered, which are (i) airspeed of 0 m/s with a pitch angle of 45°, (ii) airspeed of 5 m/s with an angle of attack of 22.5°, and (iii) airspeed of 7 m/s with an angle of attack of 0°. The attitude angle (angle of attack) of the vehicle is decreasing as the airspeed increases. For each flight state, the aerodynamic characteristics of the three different sweeping amplitudes (wingtip trajectories colored again in red, green, and blue) are measured through wind tunnel experiment and analyzed by CFD method.

#### Wind tunnel experiment result

The experiment is conducted directly with RoboFalcon2.0 platform, which is mounted on a six-axis load cell for force measurements in accordance with the attitudes shown in Fig. 4B. The CG of the platform is mounted in alignment with the measurement center of the load cell to facilitate analysis. The whole setup is installed in an open jet wind tunnel (see Materials and Methods). As conventionally defined, thrust is positive in the upwind direction and pitching moment is positive in the nose-up direction. For the windless state (*v*_∞_ = 0), we define positive lift to be vertical upward, and thrust is simply oriented forward and perpendicular to lift (aerodynamic coordinate system in Fig. 4B).

Figure 4C shows the line graphs of lift, net thrust, and pitching moments of the three sweeping amplitudes (wingtip trajectories) at different flapping frequencies for each measured state. The polylines therein are colored with the corresponding wingtip trajectories in Fig. 4 (A and B). RoboFalcon2.0 applies open-loop control for flapping frequencies, which cannot be accurately regulated with varying aerodynamic loads. For each measured state (combinations of different flight states and sweeping amplitudes), we have a roughly even scatter of four different flapping frequencies ranging from 2 to 6 Hz, which is sufficient to reveal the main trends of each aerodynamic force varying with flapping frequency.

The experimental results show that with the sweeping amplitude increasing, there is a relative enhancement of lift at higher flapping frequency, especially in flight state 1 (Fig. 4C, top left). The variation in sweeping amplitude, on the other hand, showed no particularly significant effect on thrust at the same flapping frequency for all flight states (Fig. 4C, middle column). As for the pitching moments, all three states showed a significant increase in pitching moment with the increase in sweeping amplitude (Fig. 4C, right column). From state 1 to state 3, the pitching moment exhibits an increasing trend. Its variation range with elevating sweeping amplitude transitions from spanning both sides of zero to a consistently positive range (Fig. 4E). We also compare the instantaneous pitching moments (state 3) between the flapping-only pattern and FSF wing motion in the Supplementary Materials (fig. S5). The FSF wing motion exhibits a significantly greater variation amplitude in pitching moment compared to flapping-only pattern.

#### CFD simulation result

CFD simulations based on incompressible Navier-Stokes (N-S) equations are performed to reveal the causations of the aerodynamic force/moment variations induced by the wing sweep in a visible manner. The wing kinematics in the simulations is constructed according to the wing mechanical design, replicating the trajectories of the wing joints and tips (see Materials and Methods for details). The geometry has captured the main characters of the wing surface.

Figure 4D compares the flow structures of the wing with maximum and minimum sweeping amplitudes at the middle-downstroke occasion for three different states. It can be observed that the sweeping motion could enhance the leading-edge vortex (LEV) of the wing, especially when the airspeed is low. The strength of the LEV can be inferred from the local pressure level, as a swirling vortex always induces a low-pressure zone, and the stronger the vortex is, the lower the local pressure gets. The enhanced LEV thus generates more aerodynamic loads. This could be a main reason for the lift enhancement induced by the wing sweep mentioned above.

Figure 4F, top part, displays the surface pressure distributions of the wings with different sweeping amplitudes at four downstroke occasions for state 1, and the bottom part shows the experimental and simulated values of lift and pitching moment for this state. The larger and heavier blue zones around the leading edge of the sweeping wing are right induced by the enhanced LEV. Moreover, the sweeping motion has greatly increased the planform wing area ahead of the pitching line (traversing the CG), which would correspondingly move the center of pressure (CP) ahead. Figure 4G compares the location history of the CP of the platform with the different sweeping amplitudes in Fig. 4F. We can see that the CP with maximum sweeping amplitude (red line) is ahead of that with minimum amplitude (blue line) in later stage of downstroke and earlier stage of upstroke. The forward movement of the CP can enlarge the rotate arm of the aerodynamic loads. The enhanced vortex-related force around the wing leading edge along with the prolonged rotating arm hence collaboratively result in a larger pitching moment for the wing with a larger sweeping amplitude over the one without.

This lift enhancement and pitching-moment variation brought by wing sweeping imply that it is favorable for pitch control during low-speed takeoff phase, in which the control surface (e.g., tail elevator) is substantially inefficient. This then inspires an unexplored pitch control strategy for RoboFalcon2.0.

### Bird-style takeoff simulation

To investigate the pitch control ability of reconfigurable sweeping amplitude applied in FSF wing motion during takeoff or low-speed flight state, we construct the actuation mechanism model of RoboFalcon2.0 based on physics engine MuJoCo (*43*) and simulate its wingbeat pattern and reconfigurability of kinematic parameters. An aerodynamic estimation model for the wing is built using wind tunnel data and the strip theory method adapted to FSF wing motion (see Materials and Methods), which is used as the force input for the real-time MuJoCo simulation. The model is able to effectively reproduce the main characteristics of the wind tunnel experimental results for each aerodynamic force corresponding to the flight states in Fig. 4B (see the Supplementary Materials).

**Fig. 5.**
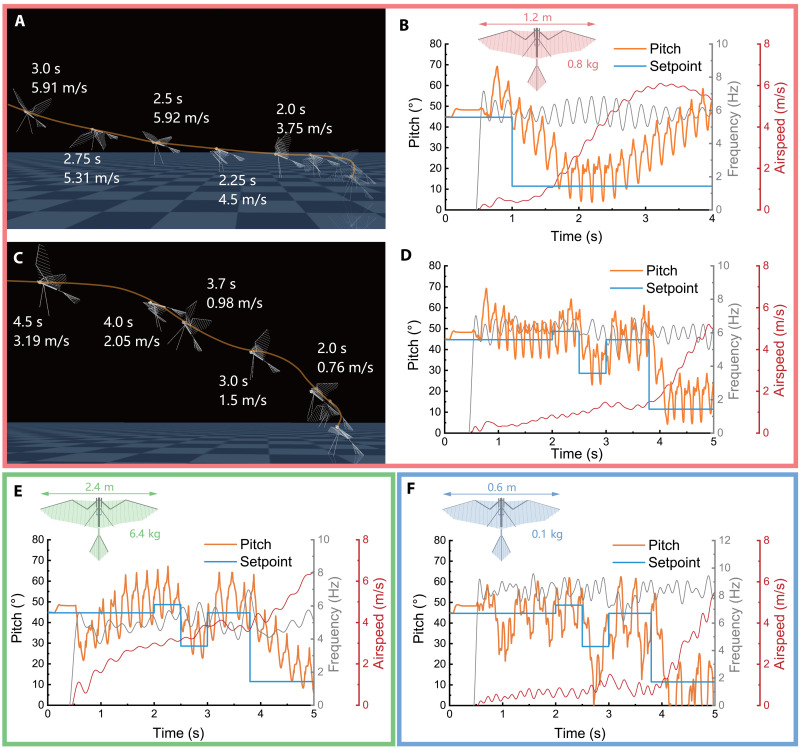
Dynamic simulation for bird-style takeoff using strip theory method in MuJoCo. (**A**) Typical bird-style takeoff process simulation, labeled with time, airspeed, and flight trajectory. The trajectory shows an upward curve as the robot is accelerated. (**B**) Time evolution of pitch angle, pitch setpoint, airspeed, and flapping frequency from takeoff simulation in left. The pitch angle diverges from setpoint after 2 s. (**C**) Low-speed flight simulation for quasi-hovering attempts. Airspeed is lower than 3 m/s within 4 s. (**D**) Time evolution of pitch angle, pitch setpoint, airspeed, and flapping frequency from low-speed flight simulation in left. The pitch follows the setpoint well under the low-speed condition. (**E**) Time evolution of pitch angle, pitch setpoint, airspeed, and flapping frequency for the twice scaled-up model performing low-speed flight simulation. The pitch setpoint is same as in (D). (**F**) Time evolution of pitch angle and pitch setpoint for the scaled-down model performing low-speed flight simulation. The pitch setpoint is same as in (D).

We present a bird-style takeoff in simulation to demonstrate the pitch control ability of reconfigurable sweeping amplitude. The simulation applies single-loop proportional-integral–derivative (PID) control on pitch and roll for the flying robot to ensure attitude stability in takeoff process. Figure 5A shows the flight trajectory of the robot takeoff simulation, which is also labeled with the airspeed and time (see movie S4). At the beginning, the robot uses a support leg to keep its body standing on the ground at about 45° elevation angle, and the pitch angle setpoint is the same as the pitch angle at this moment. The robot then takes off using the FSF wing motion with sweeping amplitude adjustment. One second later, the pitch setpoint is reduced to 10°, and the robot is gradually leaned forward affected by sweeping amplitude change, eventually transitioning to level flight. Figure 5B presents the time evolution of pitch angle and pitch setpoint during takeoff process. As we can see, the pitch angle remains stably around setpoint during the initial two or three wingbeat cycles, and the robot leaves the ground and ascends with a maintained attitude. When the pitch setpoint is reduced to 10, the pitch angle gradually decreases and converges to near 10° at 2 s. Subsequently, as the flight speed increases, the pitch angle moves away from the setpoint (pitch angle increases) and diverges.

Furthermore, even if the robot does not have effective yaw control strategy, when ensuring that there are no additional perturbations during flight, we can almost achieve an attitude-controlled bird-style hovering for a period of time in the simulation (Fig. 5C and movie S3). Figure 5D shows the simulation data of the robot maintaining a quasi-hovering flight, where the robot’s pitch angle varies slightly around 45° following pitch setpoint, while the airspeed does not exceed 3 m/s. This result shows that the pitch angle in the low-speed quasi-hovering flight (Fig. 5C; 2.0 to 3.7 s) can be controlled steadily through a simple closed-loop control system, which demonstrates that the use of sweeping amplitude adjustment for bird-style takeoff pitch control is a successful control strategy.

Because the pitching moment of flight state 1 in the wind tunnel experiment covers a range including 0 N·m (equilibrium state) and is generally linear with sweeping amplitude (Fig. 4E; state 1), the simulation results for low-speed flight in Fig. 5 (C and D) are reasonable. However, the wind tunnel test data also show that, at higher airspeeds, the head-up moment is enhanced and covers a range higher than 0 N·m (Fig. 4E; state 2 and state 3). Our aerodynamic model used in the simulation, despite discrepancies, still reproduces the phenomenon of enhanced head-up moment at increased airspeed. This phenomenon implies that the flapping wings of RoboFalcon2.0 are unable to generate sufficient head-down moment when the forward flight speed is too high, and, therefore, the pitch control fails, which explains the head-up divergence of pitch attitude in the later stage of takeoff simulation. Configuration of the robot’s CG in the simulation with reference to the low-speed state (which is exactly the CG position shown in Fig. 4 that is aligned with the load cell for pitching-moment measurement) results to the cases illustrated in Fig. 5 (A to D). Instead, to perform pitch control at higher flight speed using pitching-moment variation generated by wing sweeping motion, it is necessary to configure the CG at a location suitable for that speed or to use a tail with elevator for trimmed flight. For achieving a complete takeoff from leaving ground to higher speed cruise level flight, the elevator could be a necessary configuration.

Moreover, we also present two additional low-speed flight simulations at distinct scales to preliminarily examine the control effectiveness of sweeping-adjustable FSF wing motion across different platform sizes (Fig. 5, E and F). The simulations comprised (i) a 2× scaled model (wingspan: 2.4 m, weight: 6400 g) and (ii) a 0.5× scaled model (wingspan: 0.6 m, weight: 100 g), both derived from the default model (wingspan: 1.2 m, weight: 800 g). Simulation results for the scaled-up model (Fig. 5E) indicate controllability at proportionally higher airspeeds up to 6 m/s. The flapping frequency decreased to approximately 5 Hz compared to ~6 Hz in the default model. Pitch attitude demonstrated slower convergence rates relative to the default configuration. Conversely, simulations for the scaled-down model (Fig. 5F) revealed controllable flight only below 2 m/s. Flapping frequency increased to ~8 Hz. However, pitch attitude exhibited an oscillatory pattern with two-wingbeat-period cycles around the setpoint, likely resulting from overshoot due to accelerated convergence dynamics.

### Real-world bird-style takeoff demonstration

**Fig. 6.**
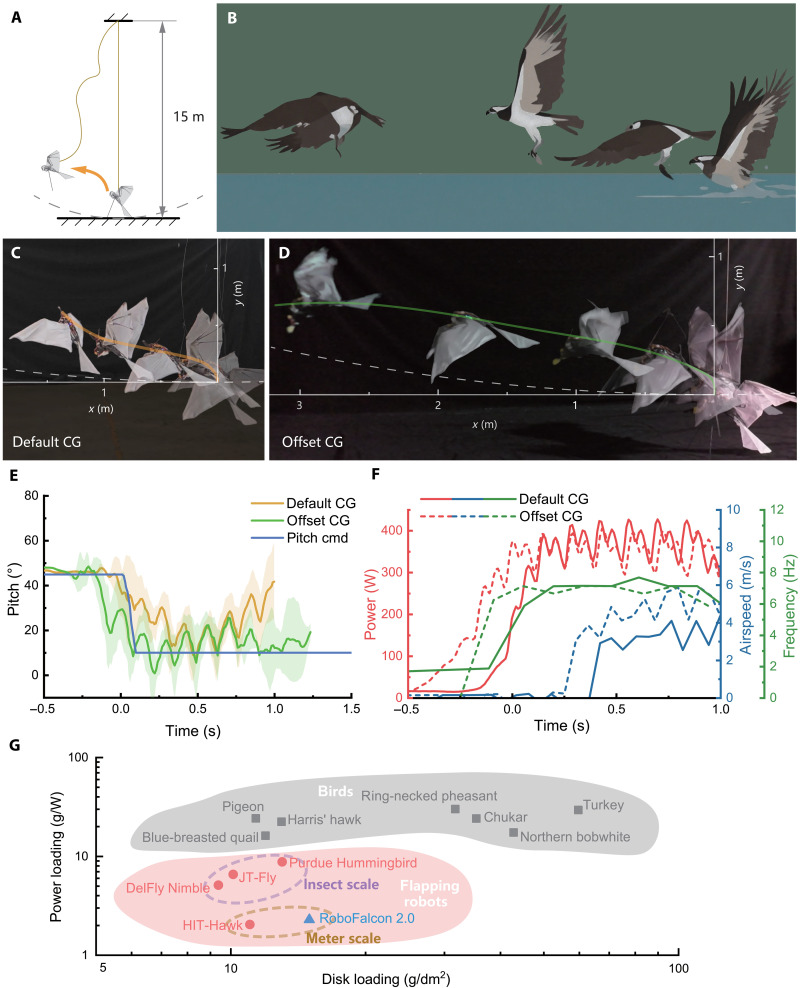
Real-world bird-style takeoffs performed by RoboFalcon2.0. (**A**) Setup for takeoff flight test. It can be considered as free flight within the spherical zone (indicated by the dashed line). (**B**) Takeoff process of osprey. (**C**) A typical bird-style takeoff performed by RoboFalcon2.0 with default CG configuration. The robot exhibits upward curved flight trajectory in later stage. (**D**) A bird-style takeoff performed by RoboFalcon2.0 with forward-offset CG configuration. The robot can accelerate continuously and maintain level flight eventually. (**E**) Time evolution of pitch setpoint and pitch angles from real-world bird-style takeoffs with default (yellow line) and offset (green line) CG configurations. The shaded area for each line represents the standard deviation from three trials. (**F**) Time evolution of power consumption, airspeed, and flapping frequency from takeoffs with default (solid line) and offset (dashed line) CG configurations. (**G**) Comparison of takeoff power loading and disk loading of RoboFalcon2.0 with other flapping robots (*32*–*34*, *36*) and birds (*45*). cmd, command.

The takeoff and pitch control capabilities of FSF wing motion are finally verified by real-world takeoff flight tests conducted on RoboFalcon2.0 prototype. The real-world experimental setup is shown in Fig. 6A, where the robot is tethered to a long lightweight string (with negligible mass), and the other end of the string is attached to the ceiling of a hangar, which is about 15 m high. The string is just about taut when RoboFalcon2.0 is standing on the ground, which means that the robot can be regarded as flying freely and untethered within the spherical space shown in Fig. 6A. If the robot flies out of the sphere, the string will be taut to limit its moving range and thus provide protection. Similar to the simulation, RoboFalcon2.0 also relies on two properly deployed support legs to stand on the ground at a 45° elevation, mimicking the pre-takeoff attitude of a bird. The robot is equipped with an onboard flight controller (based on STM32 F765) to provide closed-loop control for pitch and roll attitude, which is also used to collect data during flight, including attitude angle, power consumption, airspeed, flapping frequency, etc. (see Materials and Methods). Figure 6B gives a comparable flight snapshot of an osprey’s takeoff to demonstrate the bird’s takeoff using the FSF wingbeat pattern (movie S5). Figure 6 (C and D) shows two different CG configurations for the bird-style takeoff experiments to present the takeoff ability of RoboFalcon2.0.

Figure 6C shows a typical bird-style takeoff performed by RoboFalcon2.0 with default CG configuration (same as the wind tunnel experiment). Like the simulation, the vehicle leaves an S-shaped flight trajectory (see movie S4). During takeoff, the sweeping decoupler is at maximum sweeping amplitude in order to gain maximum lift, and the wings sweep forward like a bird during the downstroke. After takeoff from the ground, the sweeping amplitude is controlled by the flight controller to maintain the pitch attitude to follow the pitch command. The pitch angle setpoint is then reduced by a human operator (Fig. 6E, solid blue line), and the robot gradually accelerates to level flight state. As the solid yellow line shown in Fig. 6E (average of three takeoff trials), RoboFalcon2.0 is initially able to follow the control command to transition from a large pitch angle to a nearly level attitude but is unable to maintain attitude controllability after the airspeed increases beyond 3 m/s (Fig. 6F, solid blue line), similar to the simulation. On the basis of this result, we believe that the sweeping amplitude can effectively control pitch attitude in low airspeed at the beginning of a bird-style takeoff. For the trial in Fig. 6C, the flapping frequency reaches about 7 Hz (Fig. 6F, solid green line), and the maximum power consumption reaches up to 400 W (Fig. 6F, solid red line). Takeoff is not a very energy-efficient process, but for an 800-g bionic flying robot, such a flapping frequency is comparable to a natural flyer with the same scale (*44*). Compared to the simulation (Fig. 5, A and B), the airspeed during takeoff in Fig. 6C reaches, at most, 4 m/s (Fig. 6F, solid blue line), and the pitch angle diverges more quickly as the speed increases. This is probably due to a certain inaccuracy in thrust prediction by the estimation model (see the Supplementary Materials) and thus results in a lager advance ratio for the simulation.

Unlike the simulation takeoff in Fig. 5 (C and D), real-world flight experiment is always accompanied by uncertain perturbations. We could not achieve real-world hovering flight on RoboFalcon2.0 without yaw control. In addition, only using FSF wingbeat pattern cannot solve the pitch control problem perfectly for all flight states, and a tail with elevator is still required for flight trim to eliminate the adverse effect of head-up moment that increases with airspeed. RoboFalcon2.0, as a prototype to validate the unique flapping pattern, is not equipped with elevator due to the limitation of takeoff weight. However, by reconfiguring the CG with the battery mounted at a forward-extended carbon fiber rod anchored to the body frame, we demonstrate a takeoff process without attitude divergence in its later phase in Fig. 6D (movie S5). As the solid green line shown in Fig. 6E (average of three trials), RoboFalcon2.0 does not follow control command well in the initial phase but instead rapidly pitches down due to the forward-offset of CG and then accelerates. As the airspeed increases, the robot maintains a lower pitch angle without significant divergence and pitches down further in the end as a response to control command. For the trial in Fig. 6D, the airspeed of RoboFalcon2.0 reaches a maximum of 6 m/s (Fig. 6F, dashed blue line), presenting a bird-style takeoff locomotion that is more practical for flying robots. It has about the same power consumption (Fig. 6F, dashed red line) and flapping frequency (Fig. 6F, dashed blue line) as the trial in Fig. 6C.

As mentioned above, the takeoff process for meter-scale flapping-wing robot is not very energy efficient. These vehicles are typically designed for level flight as the primary flight mode, and few existing cases demonstrate self-takeoff capabilities. To better analyze the takeoff energy efficiency of RoboFalcon2.0, we compare its takeoff power loading and disk loading with those of other flapping-wing robots and birds in Fig. 6G. This comparison includes one meter-scale bird-inspired flapping robot (*36*) and three insect-scale hover-capable flapping robots (*32*–*34*). The data reveal that insect-scale robots using the coil spring–based resonant flapping mechanisms achieve significantly higher flight efficiency (*33*), whereas meter-scale flapping robots lack any energy advantage under these flight conditions. It should be noted that the bird data use aerodynamic power consumption (*45*), leading to an overestimation of their overall efficiency. Furthermore, given the differences in wing kinematics and takeoff angles between bird- and insect-scale flapping-wing robots, coupled with the limited availability of meter-scale flapping robots demonstrating self-takeoff abilities, the comparison in Fig. 6G is not rigorously comparable. Nonetheless, it still provides a useful reference for assessing the takeoff energy efficiency of meter-scale flapping-wing robots. Moreover, achieving controllable autonomous takeoff and sustained low-speed flight holds greater practical significance for expanding the flight envelope and enhancing the operational capabilities of meter-scale flapping-wing robots.

Overall, the flight test results are sufficient to confirm that RoboFalcon2.0 is able to generate enough lift to take off and gradually fly forward based on the FSF wing motion with a controlled attitude. RoboFalcon2.0 successfully disengaged from the spherical boundary defined by the tether constraint [indicated by white dashed arcs in Fig. 6 (C and D)] and ascended beyond the 0.5-m height threshold. The takeoff kinematics of the robot is very similar to the low-speed flight kinematics of flying vertebrates in Fig. 1 and generally exhibit a takeoff locomotion consistent with that of the bird shown in Fig. 6B.

## DISCUSSION

Inspired by the wingbeat patterns of flying vertebrates in slow flight and unconventional hovering, we propose a flapping-wing robot design and develop a robotic prototype, RoboFalcon2.0. It is equipped with a set of unique reconfigurable mechanisms to generate what we call the FSF wing motion. These mechanisms are able to couple the wing flapping, sweeping, and folding, following the same wingbeat period at different phases. This allows for the ability to fly at low speed like most flying vertebrates do and to perform bird-style self-takeoff processes.

Our study highlights a practical engineering solution to a rarely discussed bionic problem and applies it on a flapping-wing robot to perform slow-flight locomotion that is observed in most flying vertebrates. The wing-flapping actuation mainly relies on our previously designed CRM (Fig. 3C) (*30*), while the variant of wing sweeping and folding amplitude is realized by the sweeping and folding decoupler presented here (Fig. 3, D and E). These mechanisms follow the reconfiguration strategy (*46*) and are capable of tuning the wing motion parameters in continuous wingbeats. Owing to this, RoboFalcon2.0 retains the ability to fold the wing independently (Fig. 3F), as in the previous study, while also being able to vary stroke plane inclination by adjusting the sweeping amplitude (Fig. 3G).

The wind tunnel experiment results for the reconfigurable FSF wing motion show that a more inclined stroke plane due to enlarged sweeping amplitude produces both lift and head-up moment enhancement. CFD simulations reveal that this phenomenon can be related to the enhancement of the LEV and the forward movement of the CP. This suggests a control strategy coupling elevation force and pitch angle through sweeping amplitude adjustment, creating an underactuation system with one actuator controlling two DOFs. The MuJoCo simulation and real-world flight tests verified the effectiveness of this control strategy wherein the coupled variation of lift and pitching moment is compatible and applicable for the bird-style takeoff process.

Our mechanism design and control strategy collaboratively bring several functional advantages to RoboFalcon2.0. The reconfigurable mechanisms such as folding and sweeping decoupler reduce the burden on servos to engage the wingbeat kinematic adjustment, allowing for more lightweight and less powerful actuators to be used. This frees up the high-power main actuator to be dedicated to wing strokes driving to meet the high power demand during takeoff. In the initial stage of the takeoff process, where the demands for climb and pitch-up are coincident, an underactuation strategy that couples lift and pitching-moment control helps reduce the actuator count and simplify the takeoff operation, allowing RoboFalcon2.0 to focus on attitude stabilization and pending forward and upward passive acceleration from aerodynamic gains.

Although RoboFalcon2.0 is able to achieve pitch control under windless or low-airspeed conditions by the wings’ kinematic reconfiguration, the underactuation strategy for pitch control is not applicable to the full flight envelope, given that the pitching-moment range rises with the flight-state variation from low to high airspeed (Fig. 4C, right column). Birds and bats can adapt to different flight states from hovering to cruising with more reasonable wing sweeping ranges and tail deflection angles to optimize the position of the aerodynamic center (*17*, *18*). Birds’ takeoff dynamics are more elaborate, involving wing torsion, which occurs at the end of the downstroke, and aerodynamic contributions from the upstroke (*4*, *16*, *24*). Their tails also participate in attitude trimming and lift generation (*47*, *48*). This reveals the limitation of our current actuation mechanisms. The tail with elevator configuration would be required to extend the flight envelope to cover cruise flight. In future work, the sweeping amplitude reconfiguration can be co-operated with the elevator for pitch control in cruise flight, which would also enable the transition from cruising to low-speed flight to perform a bird-style perching landing. Examples for this are using a lightweight biomimetic feathered tail design (*49*) to adjust the aircraft’s aerodynamic center by varying the tail area or angle; using a CG shifting device, such as mechanical claws (*50*), to adapt the CG to different flight conditions; and using an elevator mechanically linked to the sweep amplitude actuator with a rational design to meet the pitching-moment compensation requirements for various flight conditions.

Nevertheless, the realization of a bird-style takeoff on a bionic flapping-wing robot through mechanical principles is still fascinating and fruitful to study. The actuation mechanisms proposed here emulate the kinematics of flying vertebrates’ low-speed flight to a greater extent within the scope of mechanical technology and reduce the attitude control complexity by deploying a proper underactuation strategy. This enables a previously unexplored robotic flapping takeoff using ventral-anterior downstroke and tucked upstroke, providing alternative perspectives and methodologies for avian-inspired robotics and avian locomotion research.

In addition, as discussed in our previous research (*30*), the design here is also an extended validation of the CRM multi-DOF reconfigurability on flapping-wing robot. It confirms the possibility of achieving a more anatomically correct controlled flapping takeoff through reasonable mechanical design. We believe this study will contribute to the development of more bionic and elaborate flapping-wing robot designs.

## MATERIALS AND METHODS

### Mechanical parts and avionics

The body frame and wing skeleton are both fabricated using carbon fiber composites. The folding decoupler is assembled from computer-numerical-control-machined aluminum parts (Fig. 3B, green). Most parts of the sweeping decoupler are fabricated using the selective laser melting metal three-dimensional (3D) printing process due to their unique geometry (Fig. 3B, blue).

**Fig. 7.**
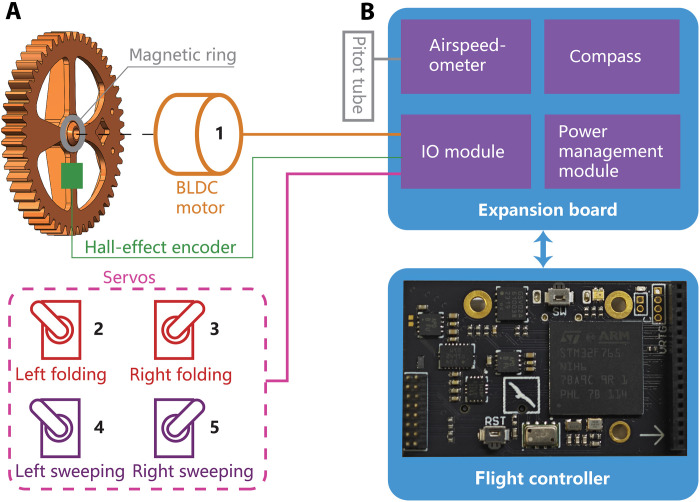
Actuators and avionics layout of RoboFalcon2.0. (**A**) RoboFalcon2.0’s actuation system has five DOFs for wings’ flapping, folding, and sweeping control. A Hall-effect encoder near the spindle of the main gear is used to record flapping data for experimental analysis. (**B**) Sensors and peripherals are integrated on an expansion board. The board transmits sensor data to the flight controller and controls commands from the flight controller to actuators. IO, input/output.

The BLDC motor (SunnySky X2216 1400 kV) shown in Fig. 3A (left; also shown in Fig. 7A) is used as the main actuator that drives the wing flapping. The actuators for folding and sweeping amplitude adjustment are high-voltage servos (GDW RS0708), which are capable of handling the alternating high-torque condition caused by wing flapping (Figs. 3B and 7A, red and purple).

The flight controller mounted on the body frame is based on the STM32 F765 microcontroller and uses open-source PX4 firmware to provide stabilization control to the flying robot (Fig. 3B, yellow, and Fig. 7B, bottom). An expansion board stacked on top of the flight controller accommodates most of the sensors, including an airspeedometer, a compass, and a power management module (Fig. 7B, top), which is used for collecting flight experimental data as well as powering the high-voltage servos. A diametrically magnetized magnetic ring (Fig. 7A, gray) is embedded around the main gear spindle, and a Hall-effect encoder (Fig. 7A, green) is mounted on the body frame near the spindle to record flapping kinematic data in flight. The Hall-effect encoder passes signals to the flight controller for logging via the on-top expansion board. Figure 7 shows in general a block diagram for the relationship of all actuators and avionics components.

### Experimental and data processing methods

The wind tunnel experiments use a low-turbulence open-jet wind tunnel located at the Northwestern Polytechnical University, which is capable of providing accurate airspeeds ranging from 5 to 20 m/s. Aerodynamic force and torque data are obtained from a six-axis load cell (ME K6D40 force/torque sensor) sampled at 1000 Hz. We use three different 3D-printed holders (small enough in size to ignore the aerodynamic interference they cause) to install RoboFalcon2.0 on the load cell to maintain the three different pitch angles in Fig. 4B without changing the mounting orientation of the load cell. Thus the six-DOF force data measured by the load cell can be directly used as the six-DOF force in wind or ground axis system of the robot without any other postprocessing.

Each flight state in Fig. 4B is measured for more than five wing-flapping cycle, allowing for cycle averaging of lift, net thrust, and pitching-moment data acquired from each state point. Cycle averaging is a common method for nontransient analysis of the flapping-wing aerodynamic variable with significant cyclicity (*51*, *52*), which helps eliminate contingencies between different flapping cycles and obtain a more accurate average value. Cycle-averaged value is calculated as follows

$$\bar{A}=\frac{\int_{0}^{nT}A\left(t\right)dt}{nT}$$ (1)

where *A* is the measured quantity and *T* is the flapping period. For the wind tunnel experiments, the maximum standard deviation of each state’s lift, thrust, and pitching moment among different flapping cycles is lower than 15 g, 10 g, and 0.02 N·m, respectively, showing that the data are collected under continuous and stable flapping condition.

### CFD method

As for the CFD simulations, the open-source software, the SU2 (Stanford University Unstructured) V7.0.7, is adopted to solve the governing equations, i.e., the 3D incompressible N-S equations (shown as Eq. 3). All simulations are run in a finite volume method framework. For spatial discretization, a second-order Jameson-Schmidt-Turkel scheme is used. For temporal discretization, a second-order dual time–stepping method is adopted. Moreover, the deforming dynamic mesh is used to realize the FSF motion of the wings. The accuracy and validity of the adopted numerical methodologies have been validated in our previous works (*53*, *54*).

∂ui∂xi=0∂ui∂t+uj∂ui∂xj=−1ρ⋅∂p∂xi+v∂ui2∂xi∂xj (2)

A hexahedron-dominating unstructured mesh with approximately 8 million cells is generated for the simulations. During each simulation, every wingbeat period is divided into 400 steps, and seven wingbeats are run. Results of the final simulated wingbeat are taken for field visualization and analysis. Moreover, both time-resolution and grid-resolution studies have been performed before formal simulations to ensure numeric convergency. The results of time-resolution and grid-resolution studies are shown in the Supplementary Materials.

The wing motion used for simulations is given by the flapping and sweeping angles along with the leading edge angles of each wing segment (humerus, radius, and handwing) as functions of time. The motion functions are presented in detail in the Supplementary Materials.

### Aerodynamic estimation model

The aerodynamic model used in bird-style takeoff simulation is based on strip theory. The strip theory (or blade element theory) is a quasi-steady method widely used for unsteady aerodynamic approximation of the flapping wing problem (*55*, *56*). Because the quasi-steady model usually requires less computational effort, it can be applied to fast real-time dynamics simulation (*57*). Some specific models are used to describe and analyze the flight of insects or insect-like flapping-wing robots with hovering capabilities (*56*, *58*), which are usually developed and calibrated only for insect-style hovering flight in the low–Reynolds number range (*Re* 100 ~ 1000) but do not predict well for level flight at higher–Reynolds number situations. Another design-oriented model is commonly used to describe the equilibrium-level flapping flight (*55*, *59*), which is a more accurate reproduction of level flight situation due to the account of leading-edge suction and vortex wake effects but cannot be applied to hovering and takeoff situations. Moreover, the above methods are only for the simple single-joint flapping wing with only flapping and twisting and cannot be applied to cases such as with RoboFalcon2.0, where the wing planform can be greatly morphed. To cope with the complex flight state and wing morphing in RoboFalcon’s takeoff simulation, we refer to previous researchers’ ideas and propose the following aerodynamic model.

**Fig. 8.**
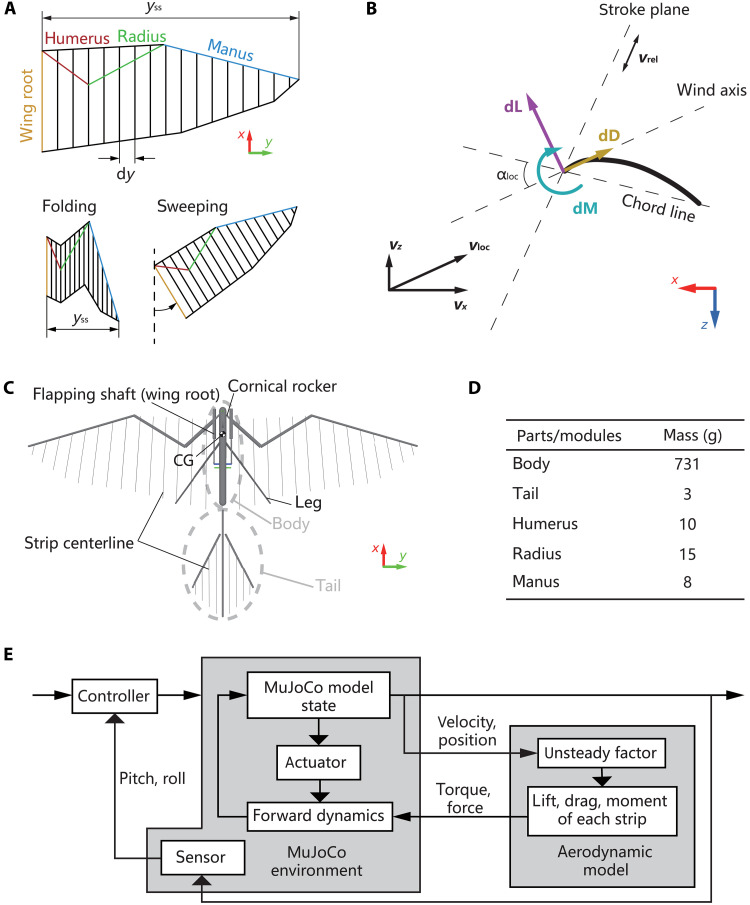
Strip theory model of FSF wing motion and MuJoCo simulation setup. (**A**) Strips layout for RoboFalcon2.0’s wing. Strip lines are parallel to the wing root when the wing is sweeping. Strip width, d*y*, varies with the wingspan when the wing is folding. (**B**) Wing section aerodynamic forces and kinematic parameters. (**C**) MuJoCo kinematic model for RoboFalcon2.0. The strips are visualized by their centerline in simulation. (**D**) Mass distribution of the kinematic model. The mass of each part is determined based on the real-world RoboFalcon2.0 prototype. (**E**) Block diagram of the MuJoCo simulation.

According to the layout in Fig. 8A, the wing surface is divided into a number of strips, which are used to calculate the aerodynamic contributions of each strip at its own airspeed and angle of attack. The strips are narrow rectangles, which are evenly distributed on the centerlines of the wing spars (humerus, radius, and manus). When the wing is folding or sweeping, the centerline of the strip rectangle is always parallel to the wing-root chord line, with a length equal to the local-section chord length and a width equal to

$$dy=\frac{y_{\text{ss}}}{n_{s}}$$ (3)

where d*y* is the width of the strip, *y*_ss_ is the transient semispan, and *n*_s_ is the number of strips.

This configuration ensures that both the shape and area of the wing model can follow the morphing motion of the wing skeleton, thus reproducing the aerodynamic characteristics of the FSF wing motion. The aerodynamic force components in Fig. 8B on each strip can be expressed as

$$dF=\frac{1}{2}\rho {v_{\text{loc}}}^{2}C_{\text{us}}C_{F}\left(\alpha_{\text{loc}}\right)c\left(y\right)dy$$ (4)

where ρ is the air density, *v*_loc_ is the local airspeed of the chord reference point (usually leading edge point) in the section plane, and *C*_F_(α_loc_) is a function of the local angle of attack α_loc_, which is a fitting result of the wings’ steady wind tunnel measured data. *C*_us_ is the unsteady factor, and it reflects the degree of unsteady effect the strip undergoes.

Specifically, for the function *C*_F_(α_loc_), the steady aerodynamic forces (lift, drag, and pitching moment) of RoboFalcon’s full-span wing are measured in the wind tunnel at different airspeeds and angles of attack (−180° ~ 180°), and then, the function *C*_F_(α_loc_) is derived by surface fitting of the measured data points. The unsteady factor *C*_us_ approximately represents most of the unsteady flow information, including added mass force and vortex wake effects. According to the unsteady characteristics of flapping-wing flight, the unsteady factor is assumed to be a variable that monotonically decreases with increasing airspeed and decreasing frequency/amplitude; thus, we construct

$$C_{\text{us}}=1+C_{\text{uh}}{\text{tan}}^{-1}\left(\frac{C_{\text{ul}}v_{\text{ref}}}{v_{x}}\right)$$ (5)

where *v*_ref_ is the reference velocity, which represents the flapping speed of the chord reference point relative to the body in the stroke plane. *v_x_* is the component of the local velocity of the strip in the *x*-axis direction of the body axis system, which characterizes the speed of air flow through the stroke plane. *C*_uh_ is the unsteady-hovering coefficient, and *C*_ul_ is the unsteady level–flight coefficient. Vectors of velocity, force, etc. are indicated in Fig. 8B.

Unsteady effects of flapping-wing aerodynamics follow different mechanisms in hovering and in level flight. The unsteady factor is introduced as an empirical correction to simplify the modeling process. When airspeed is high, *v_x_* is much larger than *v*_ref_, and the unsteady factor tends to be close to 1. In this case, the model reflects more of a steady flow state for the strip. When airspeed is low, the unsteady factor tends to be close to a constant greater than 1, and the strip is more in an unsteady flow state, which is subject to a certain enhanced aerodynamic force. We can determine the parameters *C*_uh_ and *C*_ul_ by comparing the experimental data of RoboFalcon2.0 flapping at different airspeeds with the simulation data derived from the aerodynamic model without the unsteady factor.

The introduced unsteady factor in this model compromises the accuracy of transient aerodynamic forces compared to more elaborate models. However, it also avoids discussion of complex unsteady aerodynamic effects like added mass force and LEV, thus allowing RoboFalcon’s major aerodynamic characteristics to be reconstructed and applied in simulation by relying only on steady measurements of specific wings. The specific parameters and simulation results of the model are detailed in the Supplementary Materials.

### MuJoCo simulation setup

The kinematic model used for the MuJoCo dynamics simulation is shown in Fig. 8C. Its dimensional parameters, kinematic constraints, and mass distribution (Fig. 8D) are based on the real-world RoboFalcon2.0 prototype. The model is able to effectively reproduce the fold and sweep amplitude–reconfigurable FSF wing motion of RoboFalcon2.0. The strip positions used for the aerodynamic estimated model are visualized by their centerline during simulation.

Figure 8E shows the MuJoCo simulation block diagram incorporating with the aerodynamic model. In each simulation step, MuJoCo passes the kinematic data (attitude and velocity) of the wing spars (humerus, radius, and manus) to the aerodynamic model. The aerodynamic model then solves the kinematic data of the wing spars into kinematic parameters (α_loc_, *v*_loc_, and *v*_ref_) for each strip, which are used to compute the unsteady factor *C*_us_ and aerodynamic forces (differential lift d*L,* drag d*D*, and pitching moment d*M*). Last, the aerodynamic calculations are passed back to MuJoCo to participate in its forward dynamic iterations to solve for the next state.
