# Supplementary material for: Flapping-wing robot achieves bird-style self-takeoff by adopting reconfigurable mechanisms
Source: Sci Adv. 2025 Sep 3;11(36):eadx0465. doi: 10.1126/sciadv.adx0465 (PMC12407090; doi:10.1126/sciadv.adx0465)
Supplement: Supplementary file 1 — Supplementary Text Table S1 Figs. S1 to S5 Legends for movies S1 to S5 [file sciadv.adx0465_sm.pdf]

Science Advances
AAAS

Supplementary Materials for

Flapping-wing robot achieves bird-style self-takeoff by adopting reconfigurable mechanisms

Ang Chen *et al.*

Corresponding author: Ang Chen, 15754602650@163.com; Dong Xue, xuedong@nwpu.edu.cn

Sci. Adv. **11**, eadx0465 (2025)
DOI: 10.1126/sciadv.adx0465

The PDF file includes:

Supplementary Text
Table S1
Figs. S1 to S5
Legends for movies S1 to S5

Other Supplementary Material for this manuscript includes the following:

Movies S1 to S5

Supplementary Text

FSF wing motion kinematics equations for CFD simulation

The FSF wing motion used for CFD simulation are defined by flapping angle (Θ), sweeping angle (Ψ), and humerus/radius/manus leading edge angle (α , β , γ) illustrated in Fig. S1. These angles over time are given by fitting them (Fourier fitting) to the motion data of wing skeleton obtained from mechanical kinematics simulation. The motion kinematics equations are given as follows

$$
\begin{cases}
\Theta(\varphi) \;=\; -0.07475+0.06426*cos(0.9939*\varphi)-0.764*sin(0.9939*\varphi)-0.002926*cos(2*0.9939*\varphi) \\
-0.002408*sin(2*0.9939*\varphi)-0.004979*cos(3*0.9939*\varphi)-0.03047*sin(3*0.9939*\varphi); \\
\\
\Psi(\varphi)=0.1626+0.01523*cos(0.9931*\varphi)+0.1706*sin(0.9931*\varphi)-0.07796*cos(2*0.9931*\varphi) \\
+0.01318*sin(2*0.9931*\varphi)-0.004413*cos(3*0.9931*\varphi)-0.008469*sin(3*0.9931*\varphi) \\
-0.007573*cos(4*0.9931*\varphi)+0.004584*sin(4*0.9931*\varphi)-0.002559*cos(5*0.9931*\varphi) \\
-0.001185*sin(5*0.9931*\varphi); \\
\\
\alpha(\varphi)=0.069-0.1697*cos(0.9947*\varphi)-0.001994*sin(0.9947*\varphi)+0.06002*cos(2*0.9947*\varphi) \\
-0.008044*sin(2*0.9947*\varphi)-0.0002596*cos(3*0.9947*\varphi)-0.003607*sin(3*0.9947*\varphi) \\
-0.01036*cos(4*0.9947*\varphi)+0.001791*sin(4*0.9947*\varphi)+0.001915*cos(5*0.9947*\varphi) \\
-0.001285*sin(5*0.9947*\varphi); \\
\\
\beta(\varphi)=0.3209-0.2222*cos(0.9947*\varphi)+0.003041*sin(0.9947*\varphi)+0.1011*cos(2*0.9947*\varphi) \\
-0.005319*sin(2*0.9947*\varphi)-0.01463*cos(3*0.9947*\varphi)-0.0006349*sin(3*0.9947*\varphi) \\
-0.01162*cos(4*0.9947*\varphi)+0.003726*sin(4*0.9947*\varphi)+0.005135*cos(5*0.9947*\varphi) \\
-0.001887*sin(5*0.9947*\varphi); \\
\\
\gamma(\varphi)=0.5845-0.4322*cos(0.9949*\varphi)-0.006337*sin(0.9949*\varphi)+0.1213*cos(2*0.9949*\varphi) \\
-0.02378*sin(2*0.9949*\varphi)+0.0164*cos(3*0.9949*\varphi)-0.01208*sin(3*0.9949*\varphi) \\
-0.02492*cos(4*0.9949*\varphi)+0.004347*sin(4*0.9949*\varphi)-0.0007066*cos(5*0.9949*\varphi) \\
-0.001388*sin(5*0.9949*\varphi); \\
\\
\varphi=2*\pi*f*t
\end{cases}
\tag{S1}
$$

Grid-resolution study and time-resolution study for CFD simulation

Three grids, namely, Coarse, Medium and Fine, are generated for grid-resolution study. The grid sequence has a refining ratio of $\sqrt[3]{2}$ in all three orthogonal coordinate axis directions, x,y, and z, resulting in a refining ratio of approximately 2 in volume cell numbers. Table S1 lists the account of the grid numbers. Fig. S2 compares the history of lift coefficients in a wingbeat cycle obtained with different grids and time steps. The shadow zone in the figure denotes downstroke period. For

time-resolution study, three time steps, namely, $\Delta t = T/100$, $\Delta t = T/200$, and $\Delta t = T/400$, are tested.

Table S1. Grid numbers for grid-resolution study

	Coarse	Medium	Fine
**Surface grid**	44,551	78,215	118,543
**Volume grid**	4,137,055	7,864,534	15,097,399

Parameters and fitting results for the strip theory model applied in MuJoCo simulation.

The aerodynamic force components include lift (dL), drag (dD), and pitching moment) on each strip of the strip theory model are expressed as

$$\begin{cases} \mathrm{dL} = \frac{1}{2}\rho v_{\mathrm{loc}}{}^2 \mathrm{C_{us}} \mathrm{C_L}(a_{\mathrm{loc}}) c(y)\mathrm{d}y \\ \mathrm{dD} = \frac{1}{2}\rho v_{\mathrm{loc}}{}^2 \mathrm{C_{us}} \mathrm{C_D}(a_{\mathrm{loc}}) c(y)\mathrm{d}y \\ \mathrm{dM} = \frac{1}{2}\rho v_{\mathrm{loc}}{}^2 \mathrm{C_{us}} \mathrm{C_M}(a_{\mathrm{loc}}) c^2(y)\mathrm{d}y \end{cases} . \tag{S2}$$

The unsteady factor $\mathrm{C_{us}}$ that characterises the degree of unsteady effect of the strip is given by

$$\mathrm{C_{us}} = 1 + 3.2\tan^{-1}\left(\frac{0.5 v_{\mathrm{ref}}}{v_{\mathrm{x}}}\right) \tag{S3}$$

To obtain the dimensionless force coefficients ($\mathrm{C_L}(\alpha_{\mathrm{loc}})$, $\mathrm{C_D}(\alpha_{\mathrm{loc}})$, $\mathrm{C_M}(\alpha_{\mathrm{loc}})$), we statically measured the lift, drag, and pitching moments of the RoboFalcon's full-span wing model versus airspeed (6 to 12m/s) and angle of attack (-180 to 180°) in the wind tunnel. The surface fitting results of the measured data are shown in Fig. S3. According to the fitting results and Eq. 2, the three dimensionless coefficients can be expressed as follows

$$\begin{cases} \mathrm{C_L}(\alpha_{\mathrm{loc}}) = 0.8172 + 0.4545\sin(\alpha_{\mathrm{loc}} + 0.5146) + 0.4109\sin(2\alpha_{\mathrm{loc}} + 0.2233) \\ -0.1547(3\alpha_{\mathrm{loc}} + 1.627) + 0.0975\sin(4\alpha_{\mathrm{loc}} + 0.3764) + 0.1496\sin(5\alpha_{\mathrm{loc}} - 1.227)) \\ \\ \mathrm{C_D}(\alpha_{\mathrm{loc}}) = 0.5732 - 0.3223\sin(\alpha_{\mathrm{loc}} - 35.64) + 0.2519\sin(2\alpha_{\mathrm{loc}} - 7.843) \\ +0.0639\sin(3\alpha_{\mathrm{loc}} + 2.251) - 0.0386\sin(4\alpha_{\mathrm{loc}} + 1.191) - 0.0371\sin(5\alpha_{\mathrm{loc}} + 5.581)) \\ \\ \mathrm{C_M}(\alpha_{\mathrm{loc}}) = -0.0209 - 0.3978\sin(\alpha_{\mathrm{loc}} + 0.2454) + 0.1364\sin(2\alpha_{\mathrm{loc}} + 0.6146)) \end{cases} . \tag{S4}$$

The applicability of this model for RoboFalcon 2.0 was verified in MuJoCo simulation by deploying the same measurement points as the wind tunnel experiment in main text (Fig. 4, B and C). The strip is assigned with a wingbeat-coupled sinusoidal pitch motion linearly distributed along the spanwise direction (20° pitch down and 40° pitch up at wingtip) for better thrust output in the simulation. The simulation results of cycle-averaged lift, net thrust, and pitching moment are presented in Fig. S4.

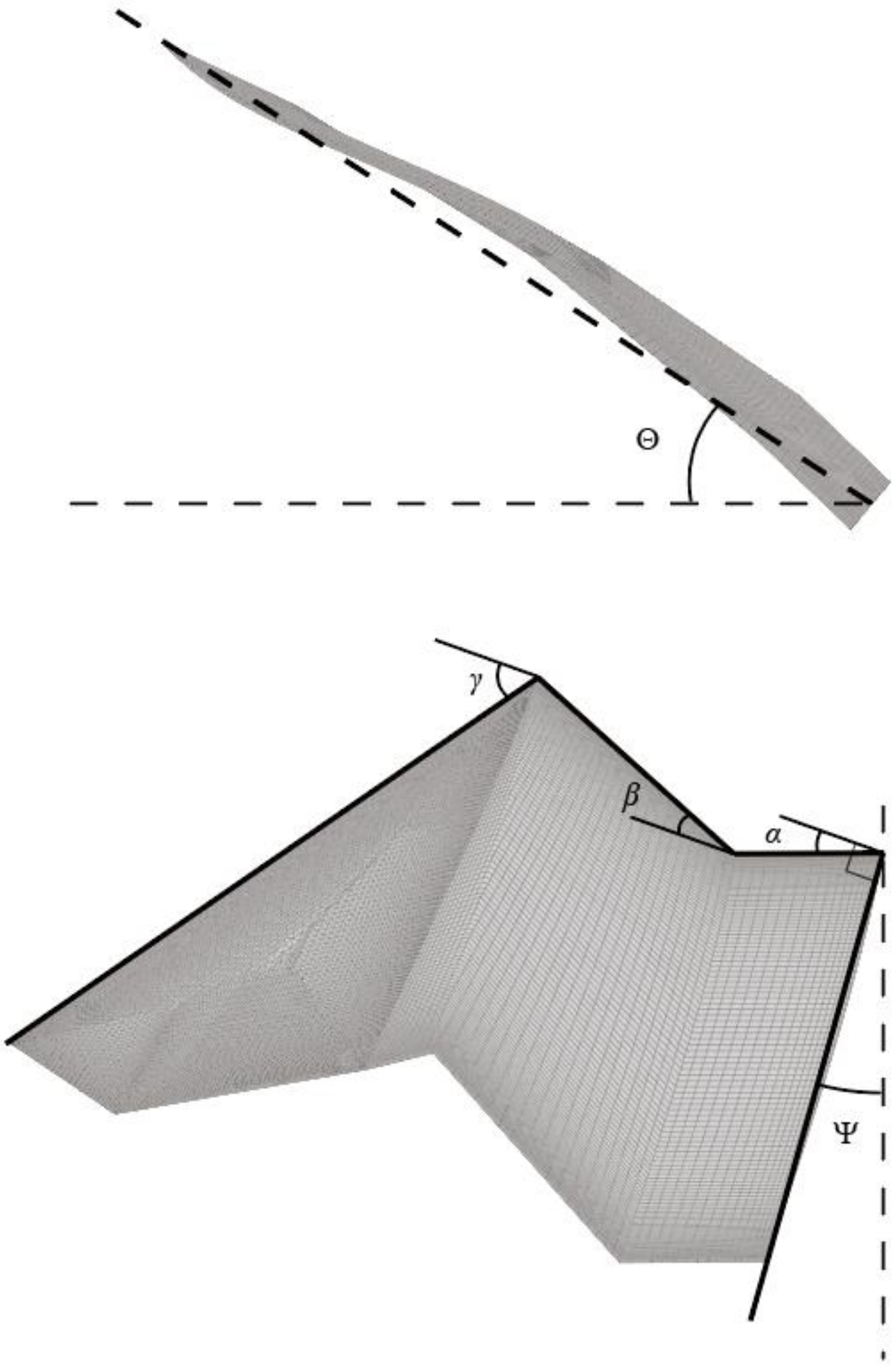

Fig. S1.

Defination of the flapping and sweeping angles and the leading edge angles of each wing segment for the FSF wing motion used in CFD simulation.

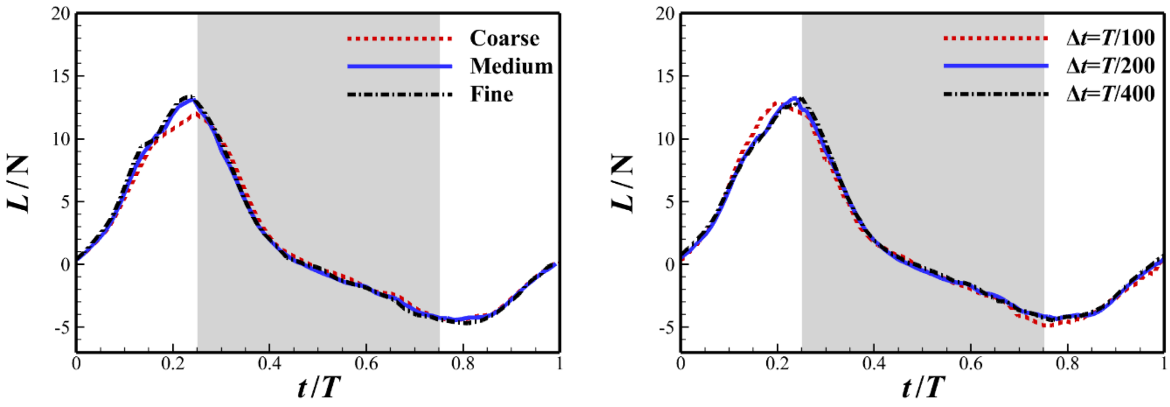

Fig. S2.
Results of grid-resolution study (left) and time-resolution study (right).

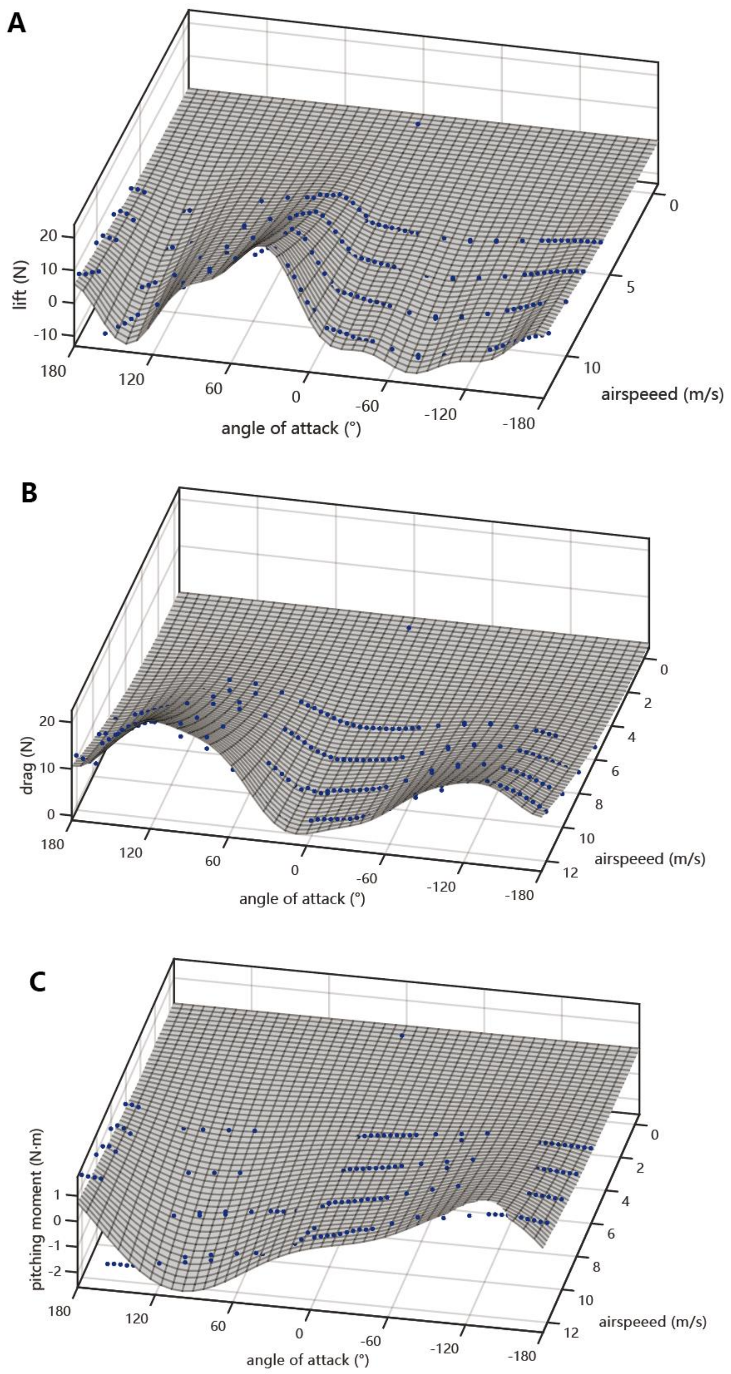

Fig. S3.

Surface fitting result of the wings' steady wind tunnel measured data. (**A**) Steady lift as a function of airspeed and angle of attack (R=0.9489, RSME=1.9948 N). (**B**) Steady drag as a function of airspeed and angle of attack (R=0.9801, RSME=0.9417 N). (**C**) Steady pitching moment as a function of airspeed and angle of attack (R=0.8785, RSME=0.4007 N·m).

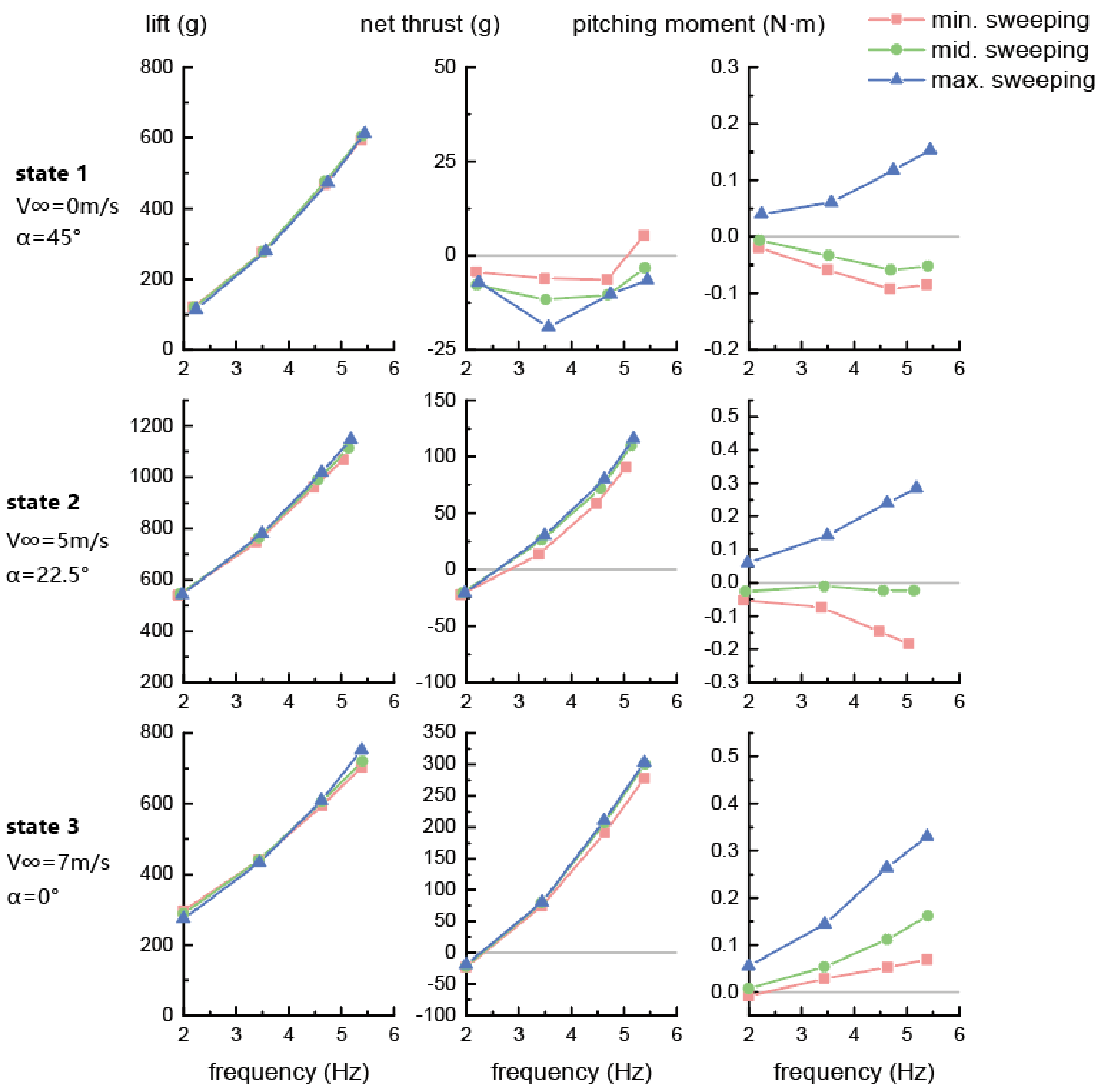

Fig. S4.

Simulation results of the strip theory model with cycle-averaged lift, net thrust, and pitching moment for different flight states.

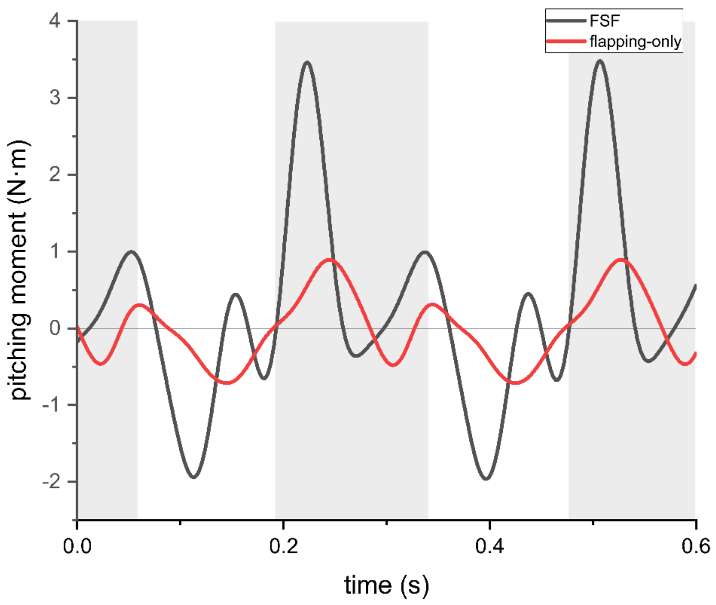

Fig. S5.

Instantaneous pitching moments of the flapping-only pattern and FSF wing motion. Measured under state 3 condition (v_∞=7m/s, α=0°), the FSF wing motion is at maximum sweeping amplitude. Downstroke phases are denoted by light grey shading in the plot. The flapping-only pattern can be achieved on RoboFalcon by removing one linkage from the Conical Rocker Mechanism, which was also adopted in our previous work (52).

Movie S1.
FSF wingbeat pattern of different flying vertebrates

Movie S2.
Mechanical principle demonstration

Movie S3.
FSF wing motion with reconfigurability

Movie S4.
Takeoff simulation

Movie S5.
Bird-style takeoff test.
